# Kaempferol and Kaempferin Alleviate MRSA Virulence by Suppressing β-Lactamase and Inflammation

**DOI:** 10.3390/molecules30204132

**Published:** 2025-10-20

**Authors:** Junlu Liu, Jingyao Wen, Jiahui Lu, Hanbing Zhou, Guizhen Wang

**Affiliations:** College of Biological and Food Engineering, Jilin Engineering Normal University, Changchun 130052, China; liujunlu@stu.jlenu.edu.cn (J.L.); wenjingyao@stu.jlenu.edu.cn (J.W.); lujiahui@stu.jlenu.edu.cn (J.L.); zhouhanbing@stu.jlenu.edu.cn (H.Z.)

**Keywords:** MRSA, β-lactamase, inflammation, secretion, anti-infection

## Abstract

Methicillin-resistant *S. aureus* (MRSA) possesses broad resistance, biofilm formation, and high virulence characteristics. Therefore, developing new therapeutic strategies against this pathogen is urgent. This work reports kaempferol (kol) and kaempferin (kin) bound to the active site of β-lactamase and interacting with key residues, thereby inhibiting its activity. In addition, kol and kin reduced the secretion of β-lactamase to the external environment, then the shielding effect of β-lactamase to β-lactam antibiotics was weakened, and finally, the bactericidal ability of ampicillin (Amp) to MRSA was enhanced. Kol and kin relieved the inflammatory responses of J774 cells induced by MRSA and improved the survival of *Galleria mellonella* (*G. mellonella*) infected by MRSA when combined with or without Amp. These data suggest that kol and kin have the potential to be developed as anti-MRSA infection agents, which would broaden the application prospects of these compounds.

## 1. Introduction

Antibiotics play an essential role in fighting *Staphylococcus aureus* (*S. aureus*) infection, which promotes the smooth development of poultry farming, food safety, and public health [1,2]. However, the inappropriate use of antibiotics also sparks the generation and development of *S. aureus* resistance [3,4]. Methicillin-resistant *S. aureus* (MRSA) shows resistance to β-lactam and many other antibiotics. MRSA has high morbidity, high mortality, and multidrug resistance characteristics [5], which has become a critical challenge in clinical treatment worldwide. Therefore, exploring and developing new anti-MRSA infection strategies has become the focus of research, and screening for natural compounds to restore the sensitivity of MRSA to antibiotics is a promising strategy.

β-lactamase is an enzyme secreted by *S. aureus* that can hydrolyze almost all β-lactam antibiotics; its encoding gene is located on the bacterial chromosome [6]. During infection, MRSA over-expresses β-lactamase, which, on the one hand, reduces the bactericidal ability of β-lactam antibiotics by hydrolyzing the lactam ring of β-lactam antibiotics. On the other hand, a large amount of β-lactamase secreted into the external environment encapsulates β-lactam antibiotics to prevent the antibiotics from entering the bacteria, which results in the inability of β-lactam antibiotics to reach the target site, which ultimately triggers the resistance of *S. aureus* to β-lactam antibiotics [3,7]. Clavulanate, sulbactam, and tazobactam were used as β-lactamase inhibitors to combat penicillin-resistant *S. aureus* infections by synergizing with β-lactam; however, the rapid emergence of bacterial resistance to the combination has created an urgent need for alternative inhibitors to restore bacterial sensitivity to β-lactam [8]. Oleanolic acid and natural tea nanoclusters (TNCs) have been identified as β-lactam antibiotic adjuvants [9,10], suggesting the feasibility of natural compounds as adjuvants of β-lactam.

Inflammation is another important aspect of *S. aureus* infection, and *S. aureus* can trigger an excessive inflammatory response in the host through several mechanisms [11,12], including the interaction between surface proteins that are anchored to the cell wall by sortase. *S. aureus* exotoxins, such as α-hemolysins (Hla), can trigger inflammation of the host by intervening with specific signal pathways [13,14,15]. In addition, immune evasion strategies and community sensing systems that help bacteria survive in the host to trigger a sustained inflammatory response [16,17]. Excessive inflammatory response not only damages host tissues but also exacerbates the *S. aureus* infection process. Therefore, inhibitors that target both β-lactamase to restore the bactericidal ability of antibiotics and inflammation to relieve *S. aureus* infection may be valuable lead compounds for the prevention and control of *S. aureus* infections.

Kaempferol (kol) and its structural analogues kaempferin (kin) are flavonoid compounds that are widely found in fruits, vegetables, and medicinal plants. Kol and kin possess a variety of biological activities and pharmacological properties related to human health, such as antioxidant, anticancer, protection of nerves, liver, and myocardium [18,19,20,21]. However, many of these functions were focused on kol; reports about kol and kin are rare. Kol has been reported to inhibit the *S. aureus* pathogenicity by targeting Hla or Sortase A [22,23]. Interactions of kol or kin with *S. aureus* β-lactamase and their role in alleviating inflammation have not been previously reported. This study reveals that kol and kin inhibited the activity of β-lactamase by direct binding; they also weakened the wrapping effect of β-lactamase to β-lactam antibiotics by inhibiting the secretion of β-lactamase to the external environment, then enhancing the bactericidal ability of ampicillin (Amp) against MRSA and inhibiting the biofilm formation. In addition, kol and kin significantly reduced the inflammatory responses of mouse macrophage cells induced by MRSA, and improved the survival of *Galleria mellonella* (*G. mellonella*) infected by MRSA.

## 2. Results

### 2.1. Analysis of the Results

#### 2.1.1. Kol and Kin Inhibited the Activity of *S. aureus* β-Lactamase by Binding to the Active Center

**Figure 1:**
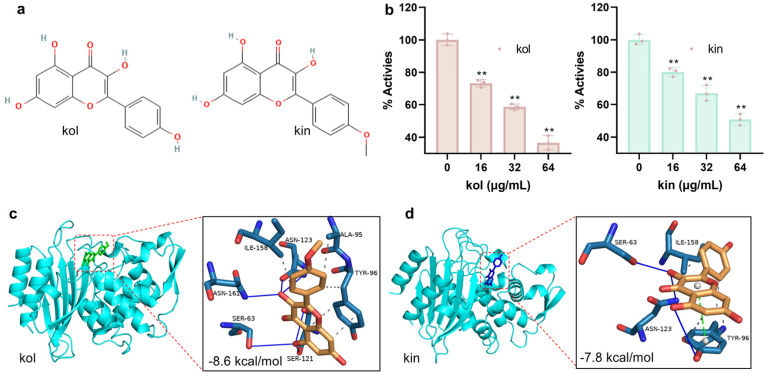
Kol and kin inhibit the activity of *S. aureus* β-lactamase by binding to the active center. (**a**) The structure of kol and kin. (**b**) The activity of β-lactamase when treated with or without tested compounds. β-lactamase protein was co-incubated with various concentrations of kol or kin, then nitrocefin was added, and samples were co-incubated. Inhibition was determined by measuring absorbance at 492 nm. Data are present as means with SD, *n* = 3, ** represents *p* ≤ 0.01. (**c**) The binding mode, the affinity, and the potential binding sites between β-lactamase and kol or kin (**d**). Protein was colored by chain (cyan), kol (colored with green) and kin (colored with blue) are shown as sticks.

The molecular structures of kol and kin are shown in Figure 1a. β-lactamase showed excellent ability to hydrolyze nitrocefin when the system did not have kol or kin, but the hydrolytic ability was reduced gradually in a kol or kin concentration-dependent manner. More precisely, the relative activity of β-lactamase was reduced to 36.60% or 50.89%, respectively, when the concentration of kol or kin was 64 µg/mL (Figure 1b), suggesting a direct interaction between kol or kin and β-lactamase exists. For further confirmation, we used oleanolic acid (a pentacyclic triterpene compound that has been disclosed as a β-lactamase inhibitor) to verify the inhibitory effect. The activity of β-lactamase decreased gradually when various concentrations of oleanolic acid were present (Table S1). To confirm the binding, docking calculation was performed, and it was found that kol and kin bound to the active pocket with the affinity of −8.6 kcal/mol and −7.8 kcal/mol (Figure 1c,d). For further confirmation, the docking poses were overlapped with β-lactamase with benzylpenicillin structure (PDB:1GHP), and we found that kol/kin bound to the binding pocket where benzylpenicillin is located (Figure S1)**.** These results indicate that kol or kin can bind to the active center of β-lactamase to inhibit its ability to hydrolyze β-lactam antibiotics.

#### 2.1.2. Kol and Kin Maintained Stable Binding with β-Lactamase

**Figure 2:**
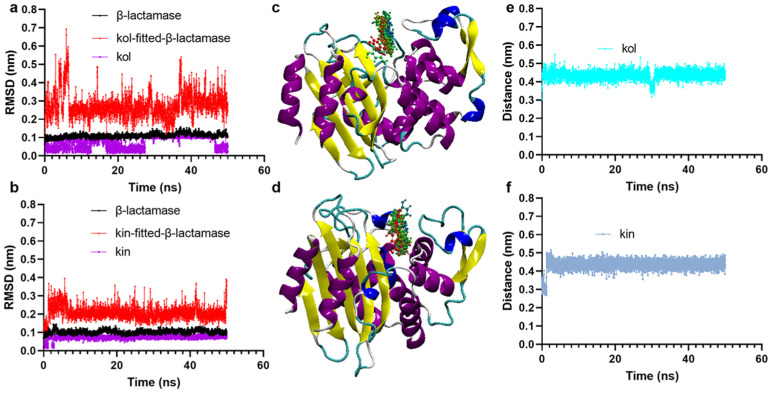
Kol and kin maintain stable binding with β-lactamase. (**a**,**b**) RMSD values of β-lactamase with kol or kin during molecular dynamics simulations. (**c**,**d**) The positions of kol or kin on the β-lactamase at different simulation times. Protein was shown as NewCartoon and colored by secondary structure, and kol and kin were shown as CPK and colored by timesteps. (**e**,**f**) The distance between β-lactamase and kol or kin against the simulation time.

To clarify the reliability of the binding, molecular dynamics simulation experiments were carried out. It was found that β-lactamase, kol, and kin maintain stable configurations during the simulation as their root mean square deviation (RMSD) values fluctuated around 0.1 nm (Figure 2a,b). The relative positions of kol or kin in β-lactamase at different simulation times also confirm the reliability of the binding (Figure 2c,d). The distance between β-lactamase and kol or kin during the simulation is approximately 0.42 nm (Figure 2e,f), suggesting the protein and its ligands maintain excellent binding.

#### 2.1.3. Hydrogen Bonds and van der Waals Interactions Were Critical for Promoting the Binding Between β-Lactamase and Kol or Kin

**Figure 3:**
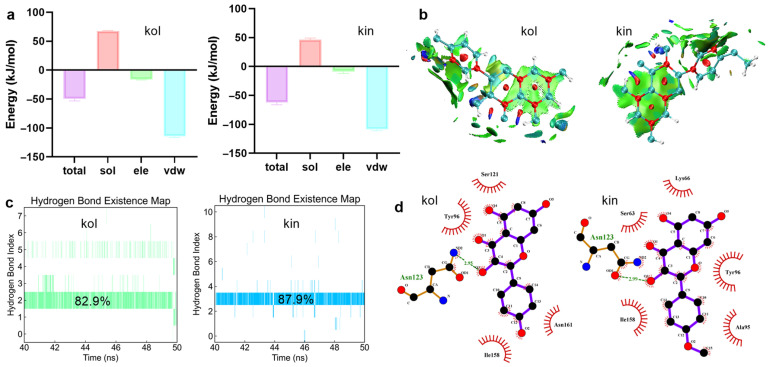
Hydrogen bonds and vdw interactions are critical in promoting the binding between β-lactamase and kol or kin. (**a**) The binding free energy between β-lactamase and kol or kin. These energies were calculated based on the MMPBSA method. (**b**) The isosurface around kol or kin. The calculation of the weak interaction between β-lactamase and kol or kin was analyzed using Multiwfn procedure. The green and blue isosurfaces indicate the vdw and hydrogen bond interaction that formed between β-lactamase and kol or kin. (**c**) The hydrogen bonds generated between β-lactamase and kol or kin, and the exact interactive atom (**d**). To present the interaction between kol, kin, and β-lactamase more accurately, hydrogen bond analysis was performed by using the last 10 ns of the equilibrium phase of the trajectory file.

To clarify the exact interactive mechanism, we detected the binding free energies between kol or kin and β-lactamase. The total binding free energy between kol or kin and β-lactamase was −49.38 kJ/mol or −62.14 kJ/mol, including van der Waals (vdw) forces of −114.49 kJ/mol for kol and −108.54 kJ/mol for kin, and electrostatic (ele) effects −16.40 kJ/mol for kol and −8.71 kJ/mol for kin (Figure 3a), suggesting vdw is critical for promoting kol or kin bound with β-lactamase, which is confirmed by the isosurface around the kol or kin (Figure 3b). Hydrogen bond analysis shows that one stable hydrogen bond exists between kol or kin and the β-lactamase complex system, with the existence of 82.9% or 87.9% (Figure 3c). The hydrogen bonds exist between ASN123 of β-lactamase and the third oxygen atom of kol or kin (Figure 3d).

#### 2.1.4. LYS66 Is Critical for the Binding Between β-Lactamase and Kol or Kin

**Figure 4:**
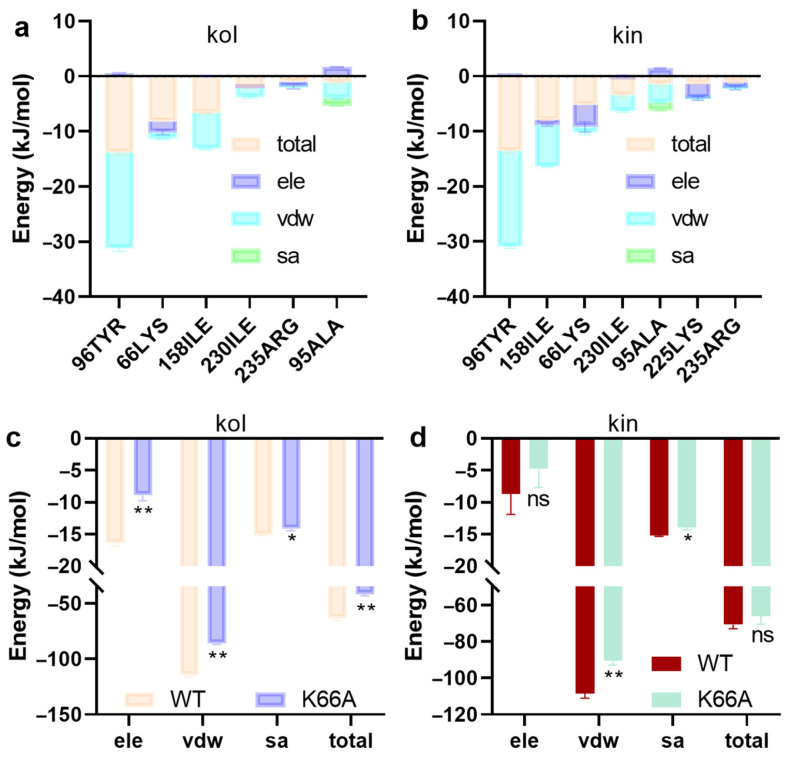
The binding free energy contribution of residues and the critical interactive residues between β-lactamase and kol or kin. (**a**,**b**) The binding free energy contribution of residues in β-lactamase for the binding with kol or kin. (**c**,**d**) The binding free energy between kol or kin and β-lactamase mutants. These energies were calculated using the MMPBSA method. The β-lactamase mutant was obtained by using SwissPdb Viewer 4.1.0. Data are present as means with SD, *n* = 3, * *p* ≤ 0.05, ** *p* ≤ 0.01, ns represents no significant.

Residue energy decomposition indicates TYR96, LYS66, ILE158, ILE230, ARG235, and LYS225 contribute more energy to the two complex systems (Figure 4a,b). For further confirmation, residue-specific mutations were performed. It was found that the binding free energy decreased when LYS66 of β-lactamase was mutated to an ALA residue (Figure 4c,d), suggesting LYS66 is the critical residue for the binding of β-lactamase with kol or kin.

#### 2.1.5. Kol and Kin Did Not Show Direct Antibacterial Activity Against *S. aureus* USA300

**Figure 5:**
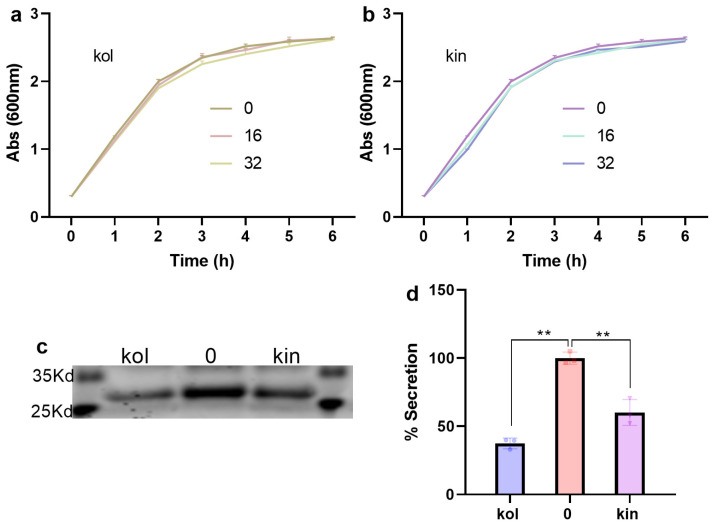
Kol or kin does not show anti-*S. aureus* USA300 characters but they inhibit the secretion of β-lactamase to the culture medium. (**a**,**b**) The growth curve of *S. aureus* USA300 when co-cultured with different concentrations of kol or kin. *S. aureus* USA300 was co-cultured with various concentrations of kol or kin with shaking. Samples were obtained every hour, and Abs_600_ values were measured. Data are present as means with SD, *n* = 3. (**c**,**d**) The secretion of β-lactamase into the culture medium when *S. aureus* USA300 was treated with or without kol or kin and the quantitative analysis. Proteins in the culture supernatant were harvested after precipitating with acetone and were separated by SDS-PAGE. The image was obtained after staining with Coomassie Brilliant Blue. The quantitative analysis was performed with Image J 1.54 g. Data are present as means with SD, *n* = 3, ** *p* ≤ 0.01.

The MIC values of kol or kin against *S. aureus* USA300 were higher than 128 µg/mL. Furthermore, *S. aureus* USA300 exhibited almost the same growth when cultured without or with various concentrations of kol or kin (Figure 5a,b), indicating that kol and kin do not possess anti-*S. aureus* characters under the test concentrations. β-lactamase was secreted to the culture medium when *S. aureus* USA300 did not receive kol or kin treatment, but the secretion was reduced to 37.50% or 60.16% when 32 µg/mL kol or kin was added (Figure 5c,d), suggesting that kol or kin inhibits the secretion of β-lactamase to the external environment.

#### 2.1.6. Kol and Kin Enhanced the Bactericidal Activity of Amp and Inhibited the Biofilm Formation of *S. aureus* USA300

**Figure 6:**
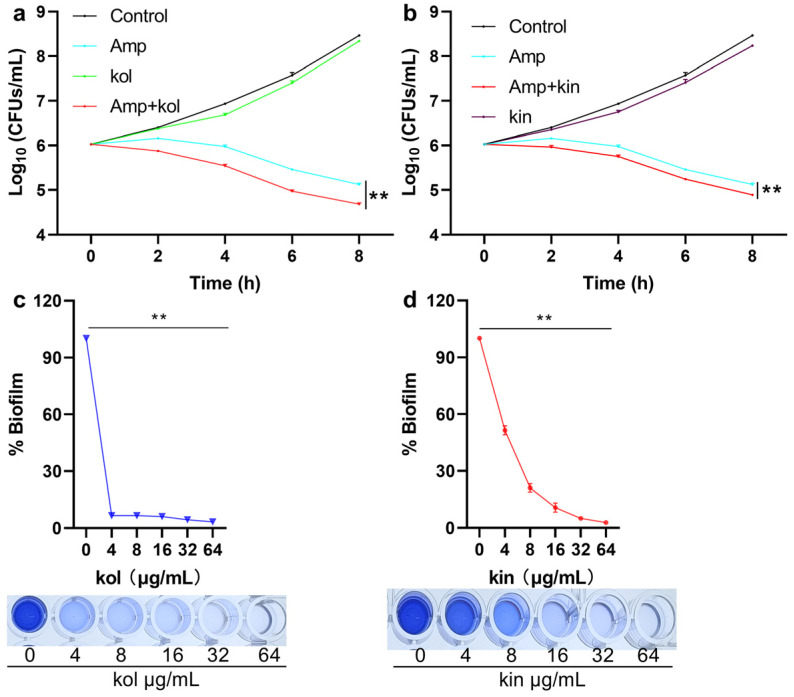
Kol or kin enhances the bactericidal ability of Amp and inhibits the biofilm formation of MRSA. (**a**,**b**) The logarithmic value of bacterial density from different treatment groups. *S. aureus* USA300 was treated with Amp, kol, kin alone, or their combination, samples were collected at two-hour intervals, clones were harvested after samples were cultured overnight on LB agar medium. Data are present as means with SD, *n* = 3, ** *p* ≤ 0.01. (**c**,**d**) The biofilm formation of *S. aureus* USA300 under various concentrations of kol or kin treatments. *S. aureus* USA300 was co-cultured with kol or kin, then samples were treated with crystal violet, and Abs_570_ values were detected after samples received acetic acid treatment. Data are present as means with SD, *n* = 3, ** *p* ≤ 0.01.

A time-dependent bacterial killing assay was carried out. It was found that *S. aureus* USA300 in the control group and the kol or kin treatment group grew normally. In the Amp treatment group, the bacteria showed stagnation in the initially four hours, and bacterial density exhibited a slight decrease at the following four hours, while, when the Amp treatment group received kol or kin treatment, bacterial density decreased gradually along with the time (Figure 6a,b), suggesting kol and kin could enhance the bactericidal ability of Amp to *S. aureus* USA300. Large amounts of biofilms were detected when *S. aureus* USA300 did not receive kol or kin treatment, but the biofilm formation sharply decreased to 6.66% when 4 µg/mL kol was added (Figure 6c). The biofilm reduced to 51.56% for 4 µg/mL kin, and the biofilm formation reduced to 10.77% or 5.03% separately when the concentration of kin reached 16 or 32 µg/mL (Figure 6d), suggesting that kol and kin could inhibit the formation of *S. aureus* USA300 biofilm.

#### 2.1.7. Kol and Kin Alleviated the Inflammatory Response of Mouse Macrophages

##### Induced by *S. aureus* USA300

**Figure 7:**
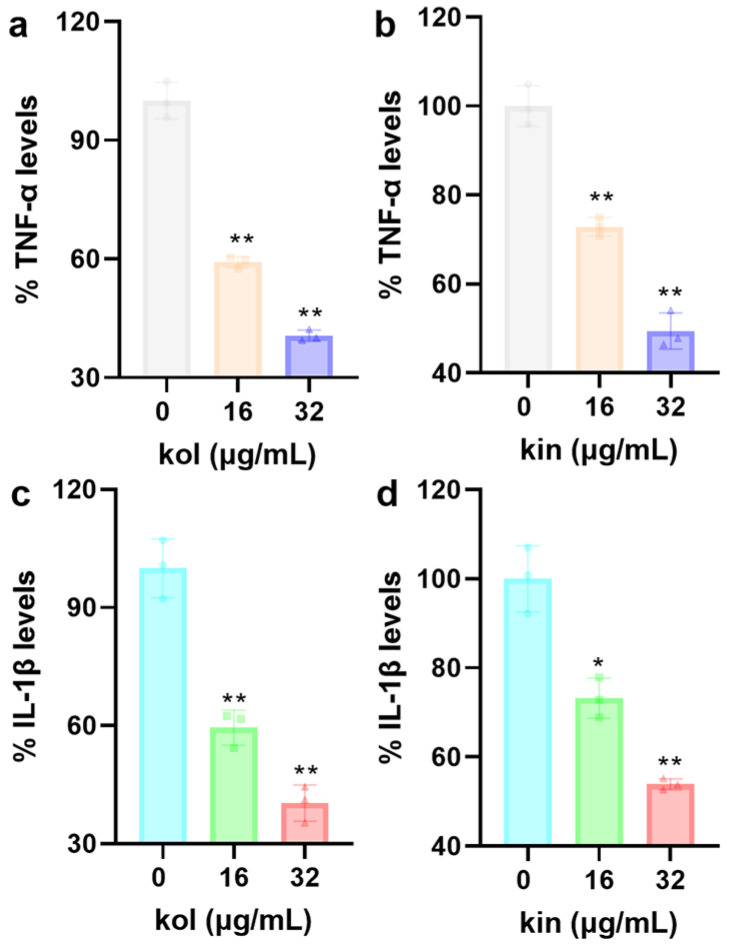
Kol or kin reduces cytokine levels. (**a**,**b**) The levels of TNF-α or IL-1β (**c**,**d**) when J774 cells received *S. aureus* USA300 and kol or kin treatments. J774 cells were treated with *S. aureus* USA300 and kol or kin. The culture medium was collected after centrifugation, and the levels of the target cytokines were determined based on the instructions of the ELISA kit. Data are presented as means with SD, *n* = 3, * *p* ≤ 0.05, ** *p* ≤ 0.01.

The levels of TNF-α and IL−1β reached 1247.69 pg/mL and 597.83 pg/mL when J774 cells were treated with *S. aureus* USA300, which indicates the pathogen triggers cellular inflammatory response. Meanwhile, when J774 cells were treated with *S. aureus* USA300 and 32 µg/mL kol or kin, the levels of TNF-α decreased to 40.62% and 49.43% (Figure 7a,b), and the levels of IL-1β decreased to 40.36% and 52.69% (Figure 7c,d). These results suggest that kol or kin could inhibit the inflammatory response mediated by *S. aureus*.

#### 2.1.8. Kol and Kin Combined with Amp Protect *G. Mellonella* from *S. Aureus* USA300 

##### Infection

**Figure 8:**
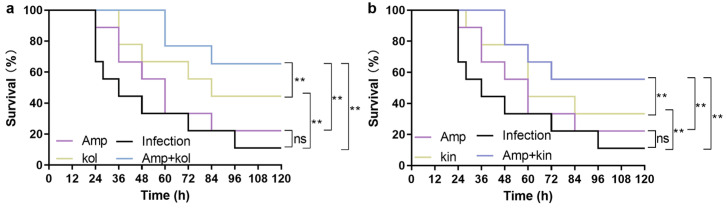
Kol or kin alone or cooperated with Amp improved the survival of *S. aureus* USA300 infected *G. mellonella*. (**a**,**b**) The survival of *G. mellonella* from different treatment groups. *G. mellonella* was treated with *S. aureus* USA300 and Amp, kol, kin alone, or their combination. The infected *G. mellonella* that received equal volume solvent treatment was defined as the infection group. Survival of *G. mellonella* under different treatment groups was statistically analyzed. Nine *G. mellonella* were arranged in each group, ns = not significant; ** *p* ≤ 0.01.

To evaluate the synergistic effect of kol or kin and Amp in vivo, we constructed an *S. aureus* USA300-infected *G. mellonella* model. The dead *G. mellonella* were detected at 24 h in the infection group and the Amp treatment group. The final survival rates for the two groups were 11.11% and 22.22%, respectively, when the infection time lasted up to 120 h, which had no significant (ns) difference (Figure 8a,b). The first death of the infected *G. mellonella* in the kol or kin treatment group occurred at 36 h and 28 h, respectively, and the final survival rates were 44.44% and 33.33%, respectively (Figure 8a,b). For the combination treatment group of Amp and kol or kin, there were no dead *G. mellonella* detected until 60 h or 48 h after infection, and the survival reached 66.67% and 55.56% (Figure 8a,b)**.** These data indicate that treatment with kol or kin alone could significantly improve the survival of *S. aureus* USA300-infected *G. mellonella*. However, when combined with Amp, the survival rate of infected *G. mellonella* clearly improved, demonstrating their potential to be developed as β-lactam antibiotic adjuvants.

## 3. Discussion

Research into the structure and function of β-lactamase has been ongoing since its discovery. To date, more than ten crystal structures of *S. aureus* β-lactamase have been identified, with or without ligands. However, reports of inhibitors of *S. aureus* β-lactamase are scarce. Kalafungin obtained from marine Streptomyces has been identified as an inhibitor of *S. aureus* β-lactamase. The authors found that kalafungin interacted with β-lactamase by forming two hydrogen bonds [24]. However, they neither reveal the effect of kalafungin on restoring the bactericidal ability of β-lactam antibiotics, nor evaluate the anti-*S. aureus* infection effects in vivo. Zhou and his partner reported oleanolic acid and its analogues as broad-spectrum inhibitors of β-lactamases to suppress the virulence of *Escherichia coli*, *Klebsiella pneumoniae,* and *S. aureus* by a direct activity inhibition manner [10]. In addition, some phenolic compounds have also been demonstrated to diminish the antibiotic resistance of *S. aureus* [25]. Here, we find that kol and kin have the potential to be developed as adjuvants of β-lactam antibiotics, as they enhanced the bactericidal ability of Amp by inhibiting the activity and the secretion of β-lactamase; on the one hand, kol and kin reduced the hydrolytic activity of β-lactamase by binding to the active center and interacting with the critical residues; on the other hand, kol and kin reduced the shielding effect of β-lactamase to β-lactam antibiotics by reducing its secretion.

The crystal structures of β-lactamase with benzylpenicillin or cephaloridine have been determined with the resolution of 1.76 Å (PDB ID: 1GHP) and 1.86 Å (PDB ID: 1GHM); SER70, GLN237, ARG244, LYS234, and SER235 have been mentioned as being involved in the hydrolytic reaction [26]. In this study, we analyzed the interactive mechanism between kol or kin and β-lactamase based on its crystal structure with a PDB ID of 6WGR. We found that these two compounds located on the active center, hydrogen bonds, and vdw are important to promoting their binding. LYS66 (LYS73 of 1GHP), ARG235 (ARG244 of 1GHP), and LYS225 (LYS234 of 1GHP) in 6WGR contributed more binding free energy, consistent with the residues identified in 1GHP that interacted with benzylpenicillin. These results were confirmed by the inhibitory effects of kol or kin against β-lactamase activity.

MRSA can form biofilms, and antibiotics face challenges in combating MRSA protected by biofilm, which further exacerbates the resistance of bacteria and results in persistent bacteria [27,28]. This leads them to establish long-term and recurrent chronic infections in the host, along with recurrent and excessive inflammation, increasing the difficulty of treatment [29]. Therefore, controlling biofilm and inflammation is expected to control recurrent chronic infections, and targeting biofilm and inflammation has become the focus of research. Some reports about anti-biofilm or anti-inflammation effects have been disclosed, including natural compounds with plant origins and their chemical derivatives. For example, coumarins and their derivatives have been found to inhibit biofilm and inflammation of *S. aureus* [30,31,32], and some chemical synthetic substances have been disclosed as inhibiting biofilm and inflammation [33,34]. Herein, kol and kin not only show anti-biofilm and anti-inflammation effects to MRSA, but also enhance the bactericidal ability of β-lactam antibiotics by targeting β-lactamase, which can significantly improve their application prospects.

As flavonoids, kol and kin have a highly similar structure; the hydrogen atom in the phenol hydroxyl group of the twelfth carbon atom of kol is replaced by a methyl group, and kol becomes kin. In this study, kol shows a better ability to inhibit the activity of β-lactamase, biofilm formation, and inflammation, and also improves the survival of *G. mellonella* that is infected by MRSA. These differences may be attributed to the phenolic hydroxyl group on the twelfth carbon atom of kol.

## 4. Materials and Methods

### 4.1. Reagents, Strains, and Cultural Conditions

β-lactamase protein was expressed and purified based on a method described previously [10] and was stored at −80 °C. Nitrocefin, which is the substrate of β-lactamase, was purchased from Shanghai Yuanye Bio-Technology Co., Ltd. (Shanghai, China). Amp was obtained from Dalian Meilun Biotechnology Co., Ltd. (Dalian, China). Kol and kin were purchased from Chengdu Purechem-Standard Co., Ltd. (Chengdu, China). The *S. aureus* USA300 strain stored in our laboratory was purchased from the American Type Culture Collection. The bacteria were cultured in Luria–Bertani (LB) medium with or without agar. The culture temperature was 37 °C under shaking or static conditions.

### 4.2. β-Lactamase Activity Inhibition Assay

β-lactamase protein (3 µg) with various concentrations of kol or kin (0, 16, 32, 64 µg/mL) in phosphate-buffered saline (PBS) buffer was co-incubated for 30 min at 37 °C, then nitrocefin (5 µg) was added, and the samples were treated for another 10 min. The absorbance value at 492 nm (Abs_492_) of each sample was obtained based on a microplate reader (Tanon, Shanghai, China). The inhibitory effects of kol or kin on β-lactamase activity were analyzed based on the absorbance values.

### 4.3. Docking and Calculation Assay

Docking and calculation assays were performed following the methods disclosed previously [35,36]. In brief, the crystal structure of β-lactamase protein (PDB ID 6WGR) was set as the receptor, and kol and kin were set as ligands. The docking box was set as 40 × 40 × 40 with a spacing of 1.0 Ångstrom (Å). AutoDock Vina 1.1.2 version [37] was used to perform the docking, and the binding was determined based on the affinity. Based on the configuration obtained by docking, molecular dynamics simulation assays were carried out to verify the reliability of binding. Amber 99SB-ildn and TIP3P were used. A 50 ns simulation assay was performed. After obtaining the trajectory files, the RMSD and the distances between ligands and β-lactamase were analyzed. The energies between ligands and β-lactamase or its mutants were calculated using the Molecular Mechanics Poisson Boltzmann Surface Area (MMPBSA) method [38]. The weak interactions between β-lactamase and kol or kin were analyzed using Multiwfn procedure [39] and the averaged NCI (aNCI) method described previously [40,41]. To confirm the critical residues, site-specific mutagenesis assays were performed using SwissPdb Viewer [42]. Briefly, Lys66 in β-lactamase was mutated to Ala by using the mutate function of the software, then the energies between the mutant and kol or kin were calculated.

### 4.4. Anti-Bacterial Properties and β-Lactamase Secretion Assay

The minimum inhibitory concentration (MIC) assays of kol or kin were performed using the method described by the Clinical and Laboratory Standards Institute (CLSI). Briefly, LB cultural medium that contained serious concentrations of kol or kin (0–128 µg/mL) in a 96-well plate was prepared, then *S. aureus* USA300 was added to each well to reach a final concentration of 5 × 10^5^ colony-forming units per milliliter (CFUs/mL). Samples were cultured at 37 °C for 24 h, and the MIC values were defined as the minimum concentration that did not support bacterial growth. For the growth curve assay, the Abs_600_ of *S. aureus* USA300 was adjusted to 0.28, then different concentrations of kol or kin (0, 16, 32 µg/mL) were added, and samples were co-cultured at 37 °C with shaking. Samples were harvested every hour to detect Abs_600_ to evaluate whether kol or kin affects the normal growth of *S. aureus* USA300. For the β-lactamase secretion assay, the culture supernatant of *S. aureus* USA300 without or with kin (32 µg/mL) or kol (32 µg/mL) was harvested. Acetone was added to each sample and treated at −20 °C overnight, and the proteins in the supernatant were harvested after centrifugation (4 °C, 12,000 rpm, 5 min). Then, sodium dodecyl sulfate-polyacrylamide gel electrophoresis (SDS-PAGE) loading buffer was added. Samples were treated at 100 °C for six minutes. Proteins were separated using a 10% SDS-PAGE gel, and an image was obtained to observe the effect of kol or kin on the secretion of β-lactamase. The quantitative analysis of β-lactamase secretion was performed with Image J 1.54 g [43].

### 4.5. Biofilm Inhibition Assay

*S. aureus* USA300 with various concentrations of kol or kin (0, 4, 8, 16, 32, 64 µg/mL) in a 96-well cell plate (2 × 10^6^ CFUs/well) were co-cultured at 37 °C for 24 h. Samples were stained with 0.1% crystal violet solution for 15 min after removing culture medium and washing with sterile PBS. Then, 33% acetic acid was used to dissolve samples, and Abs_570_ values were obtained to determine the effects of kol or kin on inhibiting the formation of biofilm.

### 4.6. Time-Dependent Bacterial Killing Assay

*S. aureus* USA300 was treated with Amp (64 µg/mL), kol (32 µg/mL), or kin (32 µg/mL) alone or Amp combined with kol or kin, and cultured at 37 °C with shaking. Samples were harvested every two hours and placed onto LB agar medium after dilution. The samples were cultured overnight at 37 °C, and the clones were harvested and analyzed to determine the effect of kol or kin on the bactericidal ability of Amp.

### 4.7. Cytokines Detection

Mouse macrophage-like cells J774A.1 in 6-well cell plates (2 × 10^6^ cells/well) were cultured in Dulbecco’s modified Eagle’s medium (DMEM) with 10% fetal bovine serum (FBS). The culture conditions were 37 °C with 5% CO_2_. The next day, the cultural medium was replaced with fresh free DMEM medium that contained *S. aureus* USA300 and different concentrations (0, 16, 32 µg/mL) of kol or kin, and the samples were co-cultured under the same conditions. The multiple of infections was five. Five hours later, the cultural medium was harvested after centrifugation (4 °C, 12,000 rpm, 5 min). The levels of tumor necrosis factor-α (TNF-α) and interleukin-1β (IL-1β) were determined using an enzyme-linked immunosorbent assay (ELISA) kit (Sangon Biotech, Shanghai, China).

### 4.8. G. mellonella Protection Assay

A total of 2 × 10^5^ CFUs of *S. aureus* USA300 were injected into *G. mellonella* (Huiyude, Tianjin, China) with a micro-syringe pump (LongerPump, Baoding, China). Kol (40 mg/kg) and kin (40 mg/kg), alone or combined with Amp (20 mg/kg), were injected into the samples after 30 min of infection. The infection group was defined as *G. mellonella* injected with *S. aureus* USA300 and received equal volume solvent treatment. The survival of the *G. mellonella* was monitored at the specified time.

### 4.9. Data Statistics and Analysis

Data obtained from three independent experiments were shown as means with standard deviation (SD). Data analysis was based on the unpaired *t*-test method that was performed in GraphPad Prism 9.5.0. The difference was determined as significant when *p* ≤ 0.05.

## 5. Conclusions

Kol and kin are bound to the active center of β-lactamase and interact with the residues that constitute the active pocket, which results in the loss of β-lactamase activity. Kol and kin did not affect MRSA growth but reduced β-lactamase secretion into the external environment, thereby diminishing its shielding effect on extracellular β-lactam antibiotics and increasing the bactericidal ability of Amp against MRSA. In addition, kol and kin inhibited the biofilm formation of MRSA and reduced the inflammation of J774 cells induced by MRSA, and improved the survival of MRSA-infected *G. mellonella*. These results suggest that kol and kin can be used as adjuvants of β-lactam antibiotics for the prevention and control of MRSA infection.

## Data Availability

Data is provided within the manuscript.

## Supplementary Materials

The following supporting information can be downloaded at: https://www.mdpi.com/article/10.3390/molecules30204132/s1, Figure S1: the structures overlap between kol/kin docking poses and β-lactamase with benzylpenicillin; Table S1: the inhibitory effects of oleanolic acid on β-lactamase activity.
