# Supplementary material for: Kaempferol and Kaempferin Alleviate MRSA Virulence by Suppressing β-Lactamase and Inflammation
Source: Molecules. 2025 Oct 20;30(20):4132. doi: 10.3390/molecules30204132 (PMC12566181; doi:10.3390/molecules30204132)
Supplement: Supplementary file 1 [file molecules-30-04132-s001.zip › molecules-3872905-supplementary.pdf]

*Supplementary Materials*

# Kaempferol and Kaempferin Alleviate MRSA Virulence by Suppressing β-Lactamase and Inflammation

**Junlu Liu, Jingyao Wen, Jiahui Lu, Hanbing Zhou and Guizhen Wang ***

College of Biological and Food Engineering, Jilin Engineering Normal University, Changchun 130052, China
* Correspondence: wanggz@jlenu.edu.cn

**Table S1 The inhibitory effects of oleanolic acid on β-lactamase activity**

| Concentrations (μg/mL) | β-lactamase activity (%) |
|---|---|
| 0 | 99.96 ± 5.76 |
| 16 | 51.23 ± 1.27** |
| 32 | 35.83 ± 2.03** |
| 64 | 28.71 ± 1.20** |

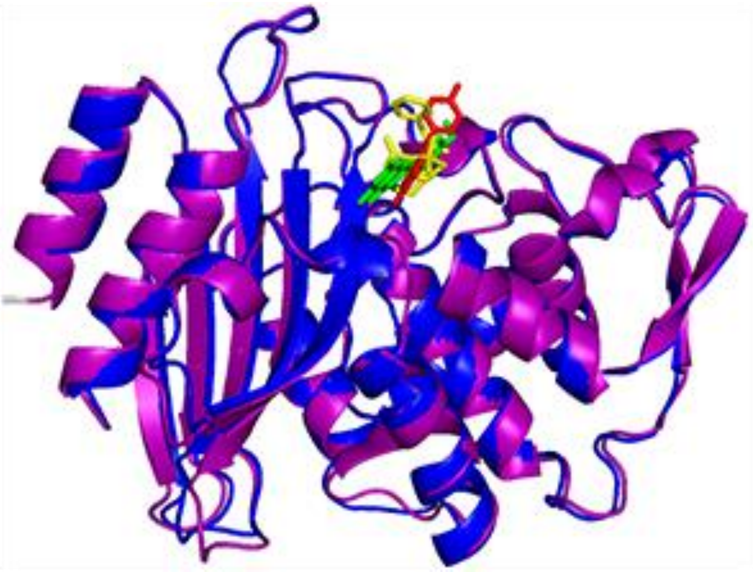

**Figure S1 The structures overlap between kol/kin docking poses and β-lactamase with benzylpenicillin.** Kol/kin was docked β-lactamase (6WGR), then the docking results were overlapped to β-lactamase with benzylpenicillin structures (PDB: 1GHP), 6WGR and 1GHP were colored by purple and blue separately, benzylpenicillin, kol and kin were colored by yellow, green and red separately.
